# Cryotherapy of Liver Tumours–A Practical Guide

**DOI:** 10.1155/1995/93283

**Published:** 1995

**Authors:** W. B. Ross, M. Horton, P. Bertolino, D. L. Morris

**Affiliations:** 1 University of New South Wales Department of Surgery The St. George Hospital Australia; 2 University of New South Wales Anaesthetic Department The St. George Hospital Australia; 3 University of New South Wales Department of Surgery The St. George Hospital Kogarah Sydney NSW 2217 Australia

## Abstract

The use of cryotherapy for the treatment of some unresectable liver tumours has been clearly established as a therapeutic option. Intra-operative ultrasound has enhanced the process by enabling the surgeon to identify hepatic lesions and to allow visualisation of the freezing process to ensure that the cryolesion will include the tumour mass. The purpose of this paper is to provide a practical guide to surgeons who wish to perform cryotherapy of liver tumours. Patient selection and anaesthetic considerations are important. The surgeon should be able to deal with the complications of cryotherapy, particularly the intra-operative haemorrhage which may arise from cracking of the hepatic parenchyma as the iceball thaws. Follow-up is based on tumour marker assay and imaging of the liver and repeat cryotherapy can be considered for selected cases.


*HPB Surgery*, 1995, Vol. 8, pp. 167–173
Reprints available directly from the publisher
Photocopying permitted by license only

© 1995 Harwood Academic Publishers GmbH
Printed in Malaysia

### Cryotherapy of Liver Tumours–A Practical Guide

W. B. ROSS[1], M. HORTON[1], P. BERTOLINO[2] and D. L. MORRIS[1,*]

[1]University of New South Wales, Department of Surgery and [2]Anaesthetic Department The St. George Hospital

The use of cryotherapy for the treatment of some unresectable liver tumours has been clearly established as a therapeutic option. Intra-operative ultrasound has enhanced the process by enabling the surgeon to identify hepatic lesions and to allow visualisation of the freezing process to ensure that the cryolesion will include the tumour mass. The purpose of this paper is to provide a practical guide to surgeons who wish to perform cryotherapy of liver tumours. Patient selection and anaesthetic considerations are important. The surgeon should be able to deal with the complications of cryotherapy, particularly the intra-operative haemorrhage which may arise from cracking of the hepatic parenchyma as the iceball thaws. Follow-up is based on tumour marker assay and imaging of the liver and repeat cryotherapy can be considered for selected cases.

KEY WORDS: Colorectal cancer cryotherapy liver hepatocellular carcinoma

### INTRODUCTION

The destruction of tumours by freezing is not new and much experimental work has been done using liquid nitrogen systems, on which current machines are based[1–3]. However, there have been considerable recent advances in the equipment used for hepatic cryotherapy, the control of freezing by intra-operative ultrasound monitoring and advances in techniques for accurate probe placement[4,5]. This procedure has largely been associated with little morbidity. The first large report of hepatic cryosurgery was in hepatocellular cancer[6]. There is now considerable evidence of reduction of serum CEA concentrations in patients with metastases from colorectal cancer[7,8] after cryotherapy and there are encouraging survival results[9,10], with some patients in complete remission[9]. Hepatic cryotherapy has in our experience been a very safe procedure but we believe that this is at least partly due to the protocols of peri-operative care which have been formulated as result of our early experience and that of others[11].

*Address for correspondence: Professor D. L. Morris
University of New South Wales, Department of Surgery
The St. George Hospital Kogarah, Sydney NSW 2217 Australia

This paper is entirely dedicated to "How to do hepatic cryosurgery".

### METHOD

### *Patient Selection*

The majority of patients treated with hepatic cryotherapy in our unit have multiple colorectal cancer metastases. A smaller number of hepatomas, neuroendocrine tumours and miscellaneous tumours have also been treated. Surgical resection remains our treatment of choice but, if the tumours are considered unresectable or the patient a poor risk for liver resection, then cryotherapy may be appropriate. Colorectal cancer metastases are considered unresectable if:- there are more than three hepatic metastases, both right and left lobes are involved, a 1 cm margin of clearance cannot be achieved (e.g. single deposits close to vital structures). The presence of extra-hepatic malignancy is also considered a contra-indication for cryotherapy but portal lymph nodes within the perfusion territory of our regional perfusion catheter may be an exception. We have considered that tumours with a diameter greater than 6 cm are unsuitable for cryotherapy, although the use of concurrent multiple probes may allow their treatment. The maximum number of hepatic deposits we have treated in one patient is 15. Patients with more than 15 hepatic deposits are offered alternative treatment where appropriate, including repeated regional perfusion with cytotoxics and selective artery chemoembolization. We have had limited experience with other tumours but melanoma in particular may not be as suitable because of the friability of tumour tissue (the single patient we treated required packing to control haemorrhage from multiple cracks in the iceball). Hepatocellular carcinoma was the first indication for hepatic cryotherapy and can be used if the tumour is not resectable[6]. It is certainly attractive in cirrhotic patients, where resection of functioning liver is avoided. Cirrhosis invariably, however, will increase the risks of cryotherapy. We have only treated two cirrhotics to date. Neuroendocrine tumours have been treated, even when there is extra-hepatic disease. Cardiovascular and respiratory function of the patients should be able to tolerate a prolonged anaesthetic (up to eight hours) and the possibility of intra-operative haemorrhage from treated lesions but we have treated many old patients with pre-existing cardiac and respiratory disease and patients of religious sects where blood transfusion is not accepted. We have not treated patients, who are jaundiced due to their liver tumour, by cryotherapy.

The majority of patients can be classified into categories ASA 1 or 11. Pugh's or Child's classification for determining surgical risk is not useful in these patients.

*Investigations*

Pre-operative investigations (Table 1) are done to confirm normal or near normal physiological and biochemical function and as baseline measurements to monitor development of intra-and post-operative complications. Two additional pre-operative arrangements need to be made, one with the Blood Bank to ensure a ready supply of compatible blood, procoagulants and platelets if required and, second, with the High Dependency or Intensive Care Unit to monitor progress and recovery of these patients for the first 48 hours.

**Table 1** Pre-operative investigations for patients undergoing hepatic cryotherapy

- Electrolytes Creatinine Urea
- Liver Function Tests Albumin
- Clotting profile
- Full blood count
- Blood glucose level
- Bone scan
- Chest x-ray
- ECG

**Table 2** Standard anaesthetic monitoring for hepatic cryotherapy

| | |
|---|---|
| ECG | $F_1O_2$ |
| $SaO_2$ | Nerve Stimulator |
| ET $CO_2$ | Urinary Catheter |

*Pre-Operative Investigations/Management*

Patients are seen pre-operatively and routine investigations ordered. Tumour marker assay is also ordered where appropriate. e.g. carcinoembryonic antigen (CEA) or alphafeto protein (AFP). Before surgery, all CT scans are reviewed with the same radiologist to re-evaluate the disease present and to plan treatment. Bowel preparation is routine, with four litres of electrolyte-polyethylene glycol solution. If a liver resection is considered to be an intra-operative option as an alternative or adjunct, then one litre of 10% dextrose is given intravenously pre-operatively. All patients (except those undergoing liver resection) receive 5,000 units of subcutaneous heparin as prophylaxis against venous thrombosis. In addition, sequential calf compression is used during the operation. Broad spectrum prophylactic antibiotics are given intravenously at the time of anaesthetic induction (Cefotaxime 1g IV). As we routinely insert a hepatic artery access port, it is important that antibiotic prophylaxis covers staphylococcus. If concomitant colonic resection is planned, patients also receive intravenous metronidazole.

*Equipment and Personnel*

We have used the L.C.S. System (Cryogenic Technology Ltd, Belper, UK) for all our cryotherapy work in Australia (Figure 1). This is a large capacity system designed specifically for hepatic cryotherapy to deliver liquid nitrogen to the tip of a triple lumen probe applied to the lesion to be frozen. Large ice-balls form in the tissue surrounding the probe. The shaft of the probe is insulated, allowing precise destruction of deeply placed hepatic lesions without significant thermal damage to overlying liver tissue. We use three probes at present: 5mm and 9 mm insulated trocar probes and a flat plate probe (Figure 2). The current machine allows simultaneous use of two probes, although four-line machines are now available and would be an advantage for hepatic work and essential if prostatic cryotherapy is also to be done. A unique thaw system has been incorporated in the specially-engineered probe to allow quicker probe detachment after freezing. Heated nitrogen gas is used in the thawing process. Liquid nitrogen (30 liters) is stored in a double walled vacuum insulated storage vessel (Dewar) with the system. This is filled from a storage tank on site under pressure before the

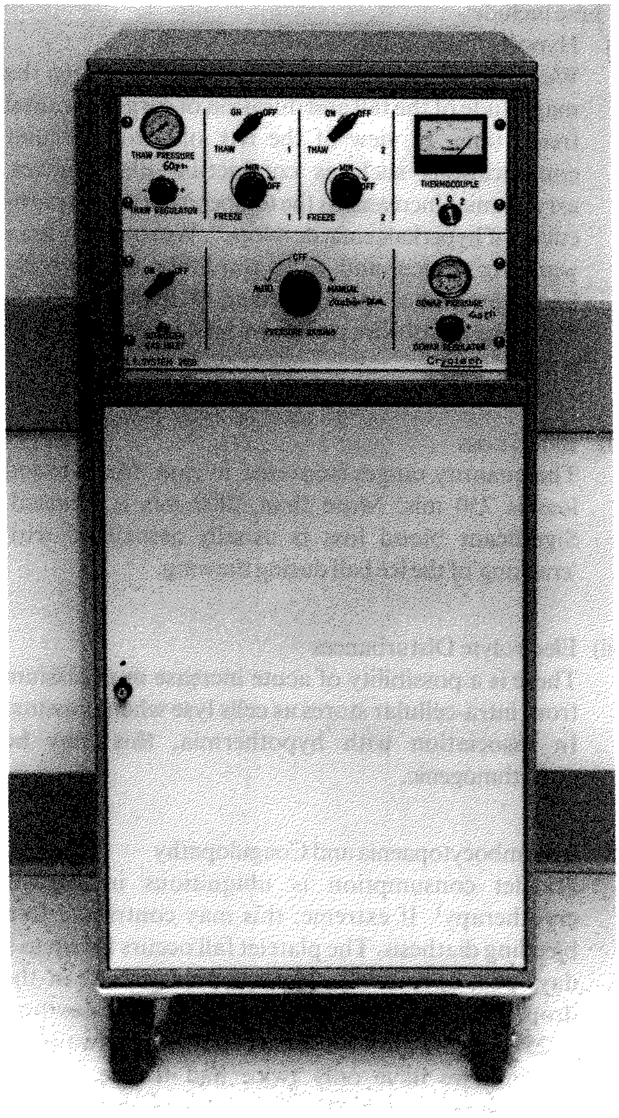

**Figure 1** Cryotech LC2000 cryotherapy apparatus.

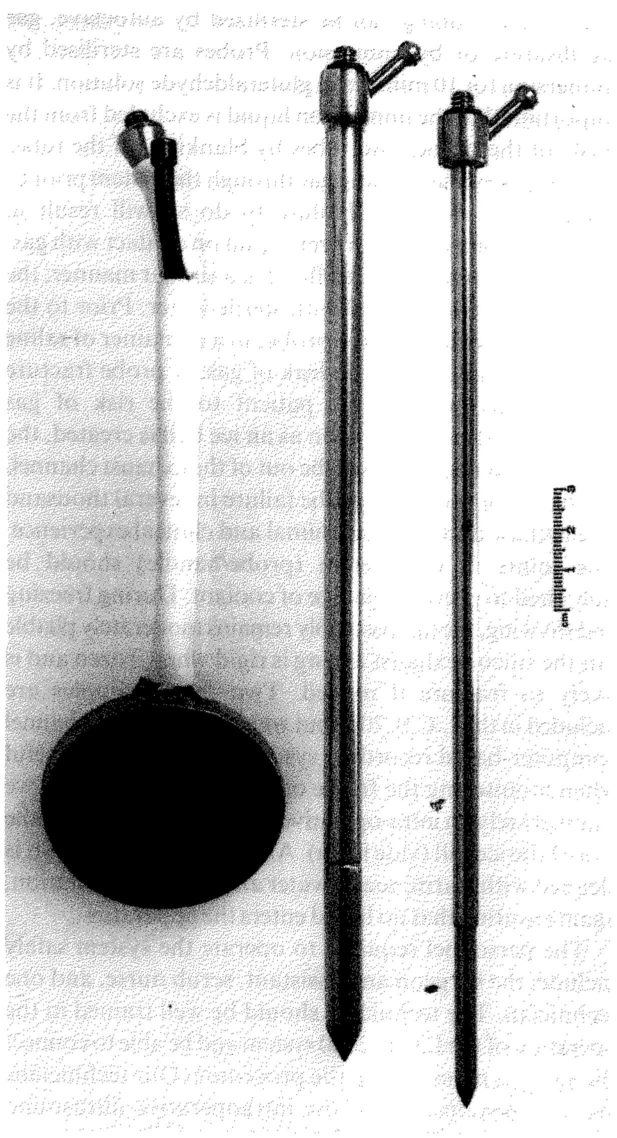

**Figure 2** Cryoprobes: Left - 5 mm trochar-tipped probe, middle - 9 mm trochar-tipped probe, right-plate.

cryotherapy procedure. The Dewar pressure is maintained at 40 p.s.i. during cryotherapy but pressures up to 55 p.s.i. can be used. Lowering the pressure to a minimum of 25 p.s.i. is a method of preserving liquid nitrogen during a long freeze for a large lesion but we believe that flow rate is probably of importance in achieving ice balls of adequate size for large livers. We use three types of probes (Figure 2). The first flat probe can be applied to surface lesions, while the two long trocar-tipped probes are inserted into the centre of lesions. The larger probe (9 cm) results in faster freezing but clearly leaves a larger defect on extraction than the smaller probe (5 mm). We now very seldom use the flat plate probe because of the limited depth of ice ball that it achieves, although it can be suitable for ovarian tumour where relatively thin plaques of tumour can be seen or treating an inadequate margin of liver resection. The freeze cycle should start at a Dewar pressure of 40 p.s.i.. This pressure is maintained for at least five minutes before it can be reduced if a long freeze cycle is anticipated. Exhaust gas is vented through a tube from the proximal part of the probe. The exhaust gas can freeze objects in close proximity. Therefore the patient, skin, viscera and assistants should be protected form exhaust tubing. The inlet and exhaust tubes are supported by a hand-held gauze sling, to avoid contact with the patient. We now use a polystyrene insulated receptacle (beer cooler) attached to the exhaust tubing for exhaust gas, to avoid damaging theatre floors. The probes and exhaust tubing can be sterilized by autoclave, gas sterilisation or by immersion. Probes are sterilised by immersion for 10 minutes in gluteraldehyde solution. It is important that the immersion liquid is excluded from the insde of the probes and tubes by blanking off the tubes with stoppers and running gas through the system prior to using liquid nitrogen. Failure to do so will result in immediate blockage by frozen liquid on contact with gas. The line assemblies are sterilised in a similar manner; the equipment is then rinsed with sterile water. Prior to the first freeze, we test run the probes in a container of saline to be sure that there is no leak of gas. A probe fracture could clearly expose the patient to the risk of gas embolism, although as soon as an ice ball is created, the path of least resistance will be out of the exhaust channel. We have not yet seen a probe failure in several thousand freeze/thaw cycles in our animal and clinical experience. The joints in the system (probe/handle) should be tightened to prevent leakage of coolant. During freezing and thawing, the line assembly remains moderately pliable but the silicon exhaust tubing is rigid when frozen and is likely to fracture if moved. Two thermocouples are included in the L.C.S. 2000 but we also use a multi-channel computer-based recording system. These may be useful when monitoring the fringe of the iceball. However, we routinely rely on intra-operative ultrasound to monitor the size of the iceball (vide infra). After use, the equipment is cleaned with warm, soapy water and sterilizing solution, again ensuring that no liquid enters the apparatus.

The personnel required to operate the system safely include: the surgeon and assistant, scrub nurse, and one technician. The technician should be well trained in the operation of the L.C.S. 2000 system and be able to connect the various pipes during the procedure. Our technicians are also responsible for the intraoperative ultrasound machine and ultrasound dissagregator.

We have had no serious incidents or malfunction in over 90 procedures in patients. The commonest technical problem we have had is leakage of liquid nitrogen between the line assembly and probe due to inadequate tightening prior to freezing and, occasionally, leaks at the joint between the L.C.S. 2000 machine and the line assembly due to inadequate fixation or, on one occasion, a cracked connector.

### *Anaesthesia for Hepatic Cryotherapy*

#### *Anaesthetic management*

Standard monitoring is applied to all patients. Other monitors are used to detect the possible intra- and post-operative sequelae of hepatic cryotherapy (Table 3).

These include:-

i) Hypothermia

We have not noted a clear relationship between the magnitude of the fall and duration of surgery or total freeze time. In view of the risk of cardiovascular complications such as conduction disturbances, arryhthmias increases as the temperature drops, particularly if hyperkalaemia develops[11]. We routinely use a warming device, and wrap the limbs with woolly bandages and a spare blanket. If hypothermia is becoming a problem, peritoneal lavage with hot saline is used.

ii) Blood Loss

The quantity ranges from case to case. Mean blood loss is 750 mls. More than 2000 mls is unusual. Significant blood loss is usually associated with 'cracking' of the ice ball during thawing.

iii) Electrolyte Disturbances

There is a possibility of acute increase in potassium from intra-cellular stores as cells lyse when thawing. In association with hypothermia, this may be arrhythmogenic.

iv) Thrombocytopaenia and Coagulopathy

Platelet consumption is ubiquitious in hepatic cryotherapy[1]. If extreme, this may contribute to a bleeding diathesis. The platelet fall occurs for up to 2 days post-operatively. The exact magnitude of the drop is unpredictable but may in individual cases drop to 25% of pre-operative values. It is almost always self limiting: we have only twice had to use platelet transfusions and both of these patients required re-operation.

v) Renal Dysfunction

Serious renal impairment is rare. Maintenance of intra-operative blood pressure, hydration, fluid resuscitation and maintenance of urine output are important measures in avoiding renal dysfunction. In our unit, diuresis is not induced with diuretics or mannitol as a

**Table 3** Supplementary anaesthetic monitors for hepatic cryotherapy

| | |
|---|---|
| • | Intra-arterial blood pressure |
| • | Central venous pressure |
| • | Oesophageal or nasopharyngeal temperature probe |
| • | Access to biochemical and haemtology labs |
| • | Any reasonable means of measuring blood loss |

routine but is used if intra-operative urine output is inadequate or if any pinkish discolouration of the urine is noted.

vi) Long Operation
Hepatic cryotherapy operations can frequently take five hours. Calf compressors, padding over pressure areas and attention to positioning of the patients should minimise venous stasis, pressure sores and neuropraxias (Table 4).

*Drugs*

If there is no evidence of hepatocellular dysfunction, then drug pharmacokinetics appear not to be altered clinically, although this has not been formally investigated during hepatic cryotherapy. It would be prudent to avoid the use of halothane and avoid the risk of decreasing hepatic blood flow and oxygen supply. Barbiturates, propofol, benzodiazepines, opiates, non-depolarising muscle relaxants have all been safely used.

*Operative Technique*

The patient is placed supine on the operating table. A right subcostal incision is made, dividing the oblique muscles and rectus abdominis. Many patients will have undergone previous colonic resection for colorectal cancer - there are often adhesions to the midline wound. These adhesions must be dissected to allow access to the liver, gall bladder and hepatico-duodenal ligament. Secondly, it is important to exclude the presence of extrahepatic disease such as local recurrence or peritoneal spread, as this would be a contraindication to treat the liver in most cases. When the decision is made to proceed, the incision may be extended by adding a left subcostal incision (roof top) depending on access, liver size and position of metastases. The lower flap of abdominal wall is reflected back and sutured down. Haemostasis of the incision and adhesion dissection should be secured. The ligamentum teres is divided and ligated. The falciform ligament is divided. Surgical access is enhanced by elevating the lower end of the sternum using a sternal lifting retractor 12. A rigid frame is placed transversely above the patient's neck, over which sterile drapes are placed. The retractor is placed under the xiphisternum and is attached by a chain to an adjustable hook on the transverse bar of the frame, while the sternum is lifted strongly by the surgeon. The operating table is tilted 20° head up. A second retractor can be used under the right costal margin and attached to the lateral part of the frame. The efficacy of this arrangement can be improved by spreading the two chains with a rod or other implement. Mobilization of the liver is important to allow full assessment of the hepatic disease and to allow access to the liver in the event of major haemorrhage, especially when treating large posterior tumours.

**Table 4** Ancillary anaesthetic equipment for hepatic cryotherapy

| 2 medium bore (14G) IV cannulae | |
|---|---|
| Calf compressors | Reflective blankets |
| Warming mattress | Warmer Humidifer |
| Fluid warmers | Foam padding |

*Intra-operative Ultrasound*

The extent of liver malignancy is assessed using a suitable intra-operative ultrasound system with sterilizable linear array or mechanical sector probes[8]. The liver is examined systematically, using the modern definitions of segmental anatomy[13]. Tumour masses are identified and their characteristics recorded: site, size, echoity (hyper/hypo-echoic). The relationship of tumour to the major vascular structures is noted.

*Application of Cryoprobes And Cryotherapy*

For small lesions on the surface of the liver, a trocar probe may be applied directly without penetration of the tumour. For larger lesions at the surface, a trocar probe is inserted into the tumour under direct vision. For lesions deeply placed within the liver substance which cannot be easily palpated, a spinal needle is directed under ultrasound guidance into the centre of the tumour. The cryotherapy probe is inserted through the liver substance, using the spinal needle as a guide both for angle and depth and visualizing the process with the ultrasound. Multiple attempts at tumour access must be avoided with the relatively large cryoprobes. In addition, the track of the probe is chosen to avoid damage to major vessels and ducts. The inlet valve on the liquid nitrogen apparatus is opened to maximum and freezing commences. At this point, gauze swabs hold the inlet and outlet tubes away from the patient's body, protecting it from cold injury. Cold gas from the exhaust should not come into contact with any object which may be damaged by cold. Theatre flooring can crack and at least one surgical assistant has been injured by cold. The iceball growth can be monitored using ultrasound. It appears very clearly as a dense black image. Freezing continues until the iceball is seen to exceed the tumour by a margin of one centimetre. During this time, the cryoprobe is supported to avoid mechanical stress on the iceball caused by movement of the liver with respiration. The average time taken to freeze a 5 cm diameter lesion is 30 minutes. Freezing time may be decreased by applying an atraumatic vascular clamp across the entire hepatic duodenal ligament. We use this method for periods of up to one hour and use this routinely for central, deep or large lesions. After achieving complete freezing of the lesion, we allow the edge of our ice ball to thaw by approximately 1 cm. In passive thawing between double freeze/thaw sequence, no gas is used. We do not allow lesions to completely thaw before re-freezing as this would take a very long time but, more importantly, is probably causally associated with the potentially fatal cryoshock syndrome of renal, pulmonary and hepatic failure with a haematological abnormality similar to disseminated intravascular coagulation. The second freeze should be allowed to go at least to the edge of the first. We then pass treated nitrogen gas (unheated gas works almost as rapidly) through the probe system allow removal of the probe well before the iceball defrosts. Ice balls are *always* allowed to completely thaw before closing the abdomen: it is impossible to assess haemostasis unless thawing is complete. The cryoprobe can be removed when rotatory movement indicated that thawing has occurred around the probe. Pre-cut fingers of oxidized celluose foam are placed in the tract vacated by the probe[14]. Liver sutures (chromic catgut) are used to control haemorrhage from the thawed iceball in about 50% of cases. Major bleeding can occur from these cracks and this can be temporarily controlled by pressure or packing. The surgeon using cryotherapy should have adequate training to deal with the more severe forms of this iatrogenic liver trauma. Our most difficult haemorrhage occurred in a soft metastatic melanoma which literally fragmented; packs were left in the abdomen and controlled this situation well. At the end of cryotherapy and thawing, a thorough check is made for haemostasis. We routinely place a suction drain above and below the liver.

### *Hepatic In-Flow Occlusion*

For large lesions, the efficacy of cryotherapy can be increased by occluding the hepatic inflow by placing a vascular clamp across the hepaticoduodenal duodenal ligament (Pringle's maneoeuvre). We have shown that this increases the zone of necrosis within the area of iceball formation[14]. Duration of clamping should not exceed one hour but it may be re-applied after a recovery period of 15 minutes.

### *Regional Cytotoxic Perfusion*

In all patients with colorectal cancer undergoing cryotherapy, we place a subcutaneous reservior and catheter to perfuse the liver. The gall bladder is removed to prevent cytotoxic cholecystitis, the right gastric artery ligated and divided and the superior aspect of the duodenum devascularised. The gastroduodenal artery is cannulated and the catheter tip fed in to sit at the origin from the common hepatic artery. It is important to use a catheter with a retaining ring so that ligatures will reliably prevent its extrusion from the artery. We check perfusion by injecting methylene blue. If unilateral perfusion is achieved, we seek the anatomical abnormality and either re-site the system or insert a second system. The catheter is heparinised.

### *Post-Operative Management*

Patients are usually managed for the first day or two on the Surgical High Dependency Unit. Mechanical ventilation may be required if the procedure was unduly long or the patient significantly hypothermic. Routine monitoring of pulse rate, blood pressure and temperature is performed. Central venous pressure and hourly urine output are measured. Pulse oximetry is routinely applied. Major potential complications include early haemorrhage, renal failure, pulmonary atelectasis, pleural effusion, subphrenic/subhepatic collections and we have seen one hepatic abscess following injection of the cryo-ablated lesion in a patient who underwent synchronous colonic resection. Most patients who have lesions near the diaphragm develop pleural effusions – rarely, these have to be drained. Peri-hepatic collections generally respond to percutaneous drainage by the radiologist. Respiratory physiotherapy is appropriate for all patients. Routine early post-operative investigation include full blood count, urea, creatinine, electrolytes, liver function tests and coagulation screen. Many patients have had previous colonic resection and have extensive intra-peritoneal adhesions which will have been dissected at the cryotherapy operation. Prolonged paralytic ileus may therefore be encountered. Post-operative pyrexia is common and a cause is often not found the cryo-necrotised liver may well be responsible. Imaging fails to demonstrate an abscess collection. Routine culture of urine, sputum, etc, although necessary is unhelpful.

### *Follow-up*

Therapeutic response is measured by monitoring tumour markers. This is measured at monthly intervals. Following cryotherapy, CEA levels fall in almost all CEA secretors. A subsequent rise in CEA level has always been predictive of local or systemic relapse. Many of these patients are investigated to assess the extent of the disease. In the absence of extra-hepatic disease, further cryotherapy or regional chemotherapy may be considered.

*Addendum 1*: Sterilization of cryoprobes: We are aware of recent concern regarding the use of glutaldehyde as a sterilizing agent. We now use ethylene oxide gas sterilization.

*Addendum 2*: The LCS 2000 has been modified to recycle liquid nitrogen. This is the LCS 3000.

### REFERENCES

1. Jacob, G., Li, A. C. K., Hobbs, K. G. T. (1984) A component of cryodestruction with excision or infraction of implanted tumour in rat liver. *Cryobiology*, **21**, 148–186.
2. Gilbert, J. C., Onik, G. M., Haddick, W. K., Rubinsky, B., Fennell, L. D. (1986) Ultrasound monitored hepatic cryosurgery. Longevity study on an animal model. *Cryobiology*, **23**, 277–285.
3. Rubinski, B., Lee, C. Y., Bastacky, J., Onik, G. (1990) The process of freezing and the mechanism of damage during hepatic cryosurgery. *Cryobiology*, **27**, 85–97.
4. Onik, G., Cooper, C., Goldberg, H. I., Moss, A. A., Rubinski, B., Christianson, M. (1984) Ultrasonic characteristics of frozen liver. *Cryobiology*, **21**, 321–328.
5. Onik, G., Kane, R., Steele, G., McDermott, W., Kheltry, U., Cody, B., Jenkins, R., Katz, J., Clouse, M., Rubinski, B., Chase, B. (1986) Monitoring hepatic cryosurgery with sonography. *American Journal of Roentology*, **147**, 665–659.
6. Zhou, X. D., Tang, Z. Y., Yu, Y. Q., and Ma, Z. C. (1988) Clinical evaluation of cryosurgery in the treatment of primary liver cancer. *Cancer*, **61** (9), 1889–1892.
7. Charnley, R. M., Thomas, M., Morris, D. L. (1991) Effect of hepatic cryotherapy on serum CEA concentration in patients with multiple inoperable hepatic metastases from colorectal cancer. *Australian and New Zealand Journal of Surgery*, **61**, 55–58.
8. Ravikumar, T. S., Kane, R., Cady, B., Jenkins, R. L., McDermott, W., Onik, G., Clouse, M., and Steel, G., Jr. (1987) Hepatic cryosurgery with intraoperative monitoring after metastatic colon carcinoma. *Archives of Surgery*, **122**, 403–409.
9. Onik, G., Rubinsky, B., Zemel, R., Weaver, L., Diamond, D., Cobb, C., Porterfield, B. (1991) Ultrasound guided hepatic cryosurgery in the treatment of metastatic colon carcinoma. *Cancer*, **67**, 901–907.
10. Morris, D. L., Horton, M. D. A'C., Dilley, A. V., Warlters, A., Clingan, P. R. (1993) The treatment of hepatic metastases by cryotherapy and regional cytotoxic perfusion. *Gut*, **34**, 1156–1157.
11. Goodie, D. B., Horton, M. D. A., Morris, R., Nagy, L. S., and Morris, D. L. (1992) The anaesthetic experience with hepatic cryotherapy for metastatic colonic carcinoma. *Anaesthesia and Intensive Care*, **20**, 491–496.
12. Goligher, J. C. (1974) A technique for highly selective parietal cell or proximal gastric vagotomy for duodenal ulcer. *British Journal of Surgery*, **61**, 337–345.
13. Bismuth, H. (1982) Surgical anatomy and anatomical surgery of the liver. *World Journal of Surgery*, **6**, 3–9.
14. Dilley, A. V., Dy, D. Y., Warlters, A., Copeland, S., Gillies, A. E., Morris, R. W., Cook, T. A. and Morris, D. L. ( 1993) Laboratory and animal model evaluation of the Cryotech LCS 2000 in hepatic cryotherapy. *Cryobiology*, **30**, 74–85.

